# Fatigue in multiple sclerosis and its relation with depression

**DOI:** 10.1192/j.eurpsy.2022.1170

**Published:** 2022-09-01

**Authors:** M. Ben Abdallah, I. Baati, A. Guermazi, F. Guermazi, S. Hentati, N. Farhat, J. Masmoudi

**Affiliations:** 1 Hedi Chaker University Hospital,, Department Of Psychiatry « A », Sfax, Tunisia; 2 Habib Bourguiba University Hospital, Department Of Neurology, Sfax, Tunisia

**Keywords:** fatigue, multiple sclerosis, Depression

## Abstract

**Introduction:**

Fatigue is one of the most common and disabling symptoms of multiple sclerosis (MS). It can be defined as a subjective lack of physical and mental energy.

**Objectives:**

To study the prevalence of fatigue in patients with MS and to determine the factors related to it, including depression.

**Methods:**

This was a cross-sectional, descriptive and analytical study, which took place in the neurology department in Sfax (Tunisia). It focused on patients with MS in remission phase. We used the Expanded Disability Status Scale (EDSS) to determine the degree of disability caused by MS, the Chalder Fatigue Scale to evaluate the fatigue, and the Hospital Anxiety and Depression Scale (HADS) to assess depressive symptoms.

**Results:**

The 93 patients included in the study had a mean age of 36.59 ± 10.69 years. The socio-economic level was low to medium in 52.7% of cases. The EDSS score ranged from 0 to 8 (median = 3.5). The total number of relapses ranged from 1 to 30 (median = 3.5). MS patients had fatigue in 72.4% of cases and depression in 26.9% of cases. Patients with a low to medium socio-economic level were more fatigued (p=0.027). High number of MS relapses, severity of disability on the EDSS, and presence of depression were associated with fatigue (p=0.014, p<10^-3^ and p=0.001, respectively).

**Conclusions:**

In MS patients, fatigue is a common symptom. Patients with reduced physical activity and greater MS-related disability have more severe fatigue, which negatively affects psychosocial functioning, increasing the risk of depression.

**Disclosure:**

No significant relationships.

